# Inhibitory effects of the combination of rapamycin with gemcitabine plus paclitaxel on the growth of pancreatic cancer tumors

**DOI:** 10.1007/s13577-024-01165-9

**Published:** 2025-01-11

**Authors:** Yuri Jobu, Miki Nishigawa, Kaoru Furihata, Mutsuo Furihata, Kazushige Uchida, Keisuke Taniuchi

**Affiliations:** 1 Department of Gastroenterology and Hepatology, Kochi Medical School, Kochi University, Kohasu, Oko-cho, Nankoku, Kochi, 783-8505 Japan; 2 Department of Pathology, Kochi Medical School, Kochi University, Nankoku, Kochi, 783-8505 Japan

**Keywords:** Pancreatic cancer, MTOR inhibitor, Rapamycin, Gemcitabine, Paclitaxel, Tumor growth, Invasion, Cell protrusion

## Abstract

We previously examined the antitumor effects of short interfering RNA nanoparticles targeting mammalian target of rapamycin (mTOR) in an orthotopic pancreatic cancer mouse model. We herein report the inhibitory effects of the mTOR inhibitor rapamycin on tumor growth in a novel established mouse model of pancreatic cancer using human pancreatic cancer cell line-derived organoids. Gemcitabine, 5-fluorouracil, and gemcitabine plus nab-paclitaxel are clinically used to treat advanced pancreatic cancer. In vitro assays showed that rapamycin strongly inhibited cell invasion, while gemcitabine, 5-fluorouracil, and gemcitabine plus paclitaxel primarily inhibited cell proliferation with minimal effects on invasion. In vivo mouse experiments demonstrated that rapamycin exhibited superior antitumor activity to S-1 (a metabolically activated prodrug of 5-fluorouracil) and another mTOR inhibitor, everolimus, while its efficacy was similar to that of gemcitabine plus paclitaxel (which was used instead of nab-paclitaxel due to concerns about allergic reactions in mice to human albumin) in a mouse model of pancreatic cancer using human pancreatic cancer cell line-derived organoids. Furthermore, the combination of rapamycin with gemcitabine plus paclitaxel exerted synergistic inhibitory effects on the growth of pancreatic cancer tumors. Although the inhibition of tumor growth was significantly stronger in everolimus-treated mice than in control mice, there were no additive anti-growth effects when combined with gemcitabine plus paclitaxel. The present results suggest that the combination of rapamycin with gemcitabine plus paclitaxel achieved the greatest reduction in tumor volumes in the mouse xenograft model and, thus, has significant clinical promise.

## Introduction

Pancreatic ductal adenocarcinoma (PDAC) is the seventh leading cause of cancer death worldwide with extremely low 5-year survival rates of 5–10% [1, 2]. Approximately 50% of patients present with metastatic or end-stage disease and 35% with localized unresectable disease. The number of treatment agents available for PDAC is limited; therefore, the development of promising novel therapeutics is highly desired. Two multidrug regimens that are first-line chemotherapy regimens for advanced PDAC patients are FOLFIRINOX [5-fluorouracil (5-FU), leucovorin, irinotecan, and oxaliplatin], which is associated with significant adverse events, such as neutropenia, and gemcitabine combined with nab-paclitaxel (GnP); however, the 5-year survival rate is still less than 5% [3, 4]. A randomized prospective study indicated that the effectiveness of FOLFIRINOX and GnP as first-line treatment options for advanced PDAC was similar based on the objective response rate (ORR; 36% in the FOLFIRINOX group and 31% in the GnP group), overall survival (OS; median OS of 11.2 months in the FOLFIRINOX group and 10.1 months in the GnP group), and progression-free survival (PFS; median PFS of 4.8 months in the FOLFIRINOX group and 4.6 months in the GnP group) [5]. Clinical trials attempted to improve the efficacy of these regimens by modifying the sequence of approved combination regimens or adding other cytotoxic agents. One of these trials tested a quadruplet regimen involving the addition of cisplatin and the 5-FU pro-drug capecitabine to gemcitabine and nab-paclitaxel. This randomized phase 2 trial showed promising findings, with more patients remaining alive and free from disease progression (31 of 42, 74%) than in the gemcitabine plus nab-paclitaxel group (19 of 41, 46%) [6]. Therefore, future studies are needed to assess the efficacy of the combination of gemcitabine and nab-paclitaxel with other agents for advanced PDAC.

AKT, a downstream effector of the v-akt murine thymoma viral oncogene homolog, phosphorylates p70 ribosomal protein S6 kinase (p70-S6K) and eukaryotic initiation factor 4E binding protein 1, leading to increases in mRNA translation and protein synthesis, ultimately promoting cell proliferation and growth [7, 8]. Previous studies reported increases in the expression of AKT and activation of mTOR in PDAC [9, 10]. The RAS-activated phosphoinositide 3-kinase (PI3K) pathway also promotes cell survival and proliferation in PDAC through its downstream effectors AKT and mTOR [11]. Our in vivo experiments using an orthotopic transplant mouse model demonstrated the crucial role of mTOR in the progression, invasiveness, and metastasis of PDAC [12]. The knockdown of mTOR using small interfering RNA nanoparticles significantly inhibited retroperitoneal and peritoneal dissemination, which improved the prognosis of mice [12]. Rapamycin, an mTOR-targeted agent, is approved for lymphangioleiomyomatosis and as an immunosuppressant for renal transplantation, but has yet to be approved for the treatment of malignancies [13, 14]. While rapamycin targets the PDAC endothelium for destruction and induces tumor vessel thrombosis, leading to the inhibition of tumor growth and metastasis [15], a phase II clinical trial (NCT00499486) reported the limited efficacy of single-agent rapamycin for advanced PDAC [16]. However, the addition of rapamycin to a gemcitabine/docetaxel regimen appeared to reverse tumor resistance in patients with metastatic PDAC [17].

Given the limited efficacy of single-agent rapamycin, future clinical trials on advanced PDAC patients need to examine the safety and effectiveness of combining rapamycin with other agents, particularly gemcitabine-based chemotherapy regimens. Everolimus, another mTOR inhibitor, is the 40-O-(2-hydroxyethyl) derivative of rapamycin, and has been approved for the treatment of tuberous sclerosis complex, breast cancer, renal cell carcinoma, and gastroentero-pancreatic neuroendocrine tumors [18]. Phase II clinical trials indicated that single-agent everolimus [19] and the combination of everolimus, cetuximab, and capecitabine [20] both exhibited minimal clinical activity and were not recommended for the treatment of advanced PDAC due to their low efficacy and/or toxicity.

We established a novel preclinical mouse model via pancreatic cancer organoid culture methods using the human PDAC cell line S2-013 [21]. This new model provides tumor xenografts that closely resemble clinical human PDAC tissue in morphology and architecture. We employed this preclinical model to investigate whether the combination of rapamycin with gemcitabine plus paclitaxel (GP) exerted stronger suppressive effects on tumor invasion and growth, thereby demonstrating the in vivo antitumor effects of this combination therapy.

## Materials and methods

### Antibodies

Anti-p70S6K (66,638-1-lg), anti-caspase-3 (19,677-1-AP), and anti-caspase-7 (27,155-1-AP) antibodies were purchased from ProteinTech (Rosemont, IL). An anti-phosphorylated p70S6K antibody (A7189) was obtained from Assay Biotechnology Company, Inc. (Fremont, CA). Anti-cleaved caspase-3 (9664) and anti-cleaved caspase-7 (8438) antibodies were supplied by Cell Signaling Technology (Danvers, MA). An anti-Ki-67/MKI67 (SP-6) antibody (NB600-1252) was purchased from Novus Biologicals (Centennial, CO).

### Cell culture

The human PDAC cell line S2-013, a subline of SUIT-2, was donated by Dr. Michael Hollingsworth at the University of Nebraska (Omaha, NE). The human PDAC cell line PANC-1 was obtained from the American Type Culture Collection (Manassas, VA). S2-013 and PANC-1 cells were maintained in Dulbecco’s modified Eagle’s medium (Gibco-BRL, Carlsbad, CA) containing 10% fetal calf serum at 37 °C. Human endothelial cells derived from human umbilical vein endothelial cells (HUVECs) and human mesenchymal stem cells (MSCs) were cultured as previously reported [21].

In selected experiments, S2-013 and PANC-1 cells were cultured with rapamycin (20 and 100 nM; TCI, Tokyo, Japan) for 48 h, everolimus (0.1, 1.0, and 10 µM; FUJIFILM Wako Pure Chemical Corporation, Osaka, Japan) for 48 h [22], gemcitabine (20 and 100 nM; TAIHO PHARMA, Tokyo, Japan) for 48 h [23], paclitaxel (20 and 100 nM; FUJIFILM Wako Pure Chemical Corporation) for 48 h, and 5-FU (20 and 100 nM; Yakult, Tokyo, Japan) for 48 h [23].

### Immunoblotting analysis of cell lysates

Immunoblotting was performed as previously reported [24]. Briefly, cell pellets were lysed using lysis buffer [Tris–HCl (pH 7.4), sodium dodecyl sulfate, mercaptoethanol, and glycerol], and protein concentrations were measured by the Bradford assay (#500–0006, Bio-Rad, Hercules, CA) using SpectraMax190 (Molecular Devices, San Jose, CA) or Bio Tek Cytation 5 (Agilent, Santa Clara, CA). Ten micrograms of protein was used along with primary antibodies at a dilution of 1:1,000 in 3% phosphoBLOCKER Blocking Reagent (AKR-103, Cell Biolabs, San Diego, CA) in TBST at 4 °C overnight. Following an incubation with appropriate secondary antibodies conjugated with horseradish peroxidase (sc-2371, sc-2385; Santa Cruz Biotechnology, Dallas, Texas) at a dilution of 1:8,000 at room temperature for 1 h, immunoreactive bands were visualized using the suitable ECL kit (GE Healthcare, Chicago, IL), ECL Plus kit (Thermo Fisher Scientific, Waltham, MA), or ECL Prime kit (GE Healthcare) according to the manufacturers’ instructions.

### In vitro* growth rate by the MTT assay*

Cells were seeded at a concentration of 5 × 10^4^ cells per well using 6-well plates. After an incubation for 24 h, cells were separately treated with the following therapeutics: rapamycin, everolimus, GP, and 5-FU, as described above in the Cell culture section. Forty-eight hours after the first addition of therapeutics, cell counting kit-8 solution (Dojindo, Kumamoto, Japan) was added to each well at a concentration of 1/10 volume, and plates were incubated at 37 °C for an additional 4 h. Absorbance was then measured at 450 nm, with 630 nm as a reference, using a Molecular Devices SpectraMax190 (Molecular Devices, San Jose, CA) or BioTek Cytation 5 (Agilent). Results were displayed as mean absorbance relative to controls (untreated or the DMSO control as indicated). The assay was conducted 3 times independently.

### Matrigel invasion assay

S2-013 and PANC-1 cells were seeded at densities of 3.5 × 10^4^ and 9.0 × 10^4^ cells per well, respectively, on 6-well plates. After an incubation for 24 h, cells were treated separately with the following therapeutics, including rapamycin, everolimus, GP, and 5-FU, as described above in the Cell culture section. A two-chamber invasion assay was used to assess cell invasion (24-well plates, membrane with a pore size of 8 µm coated with a layer of Matrigel extracellular matrix proteins; Becton Dickinson) as previously reported [25]. The assay was conducted 3 times independently.

### Confocal immunofluorescence microscopy

Immunocytochemistry was performed as previously reported [26]. Briefly, cells were treated separately with the following therapeutics, including rapamycin, everolimus, GP, and 5-FU, as described above in the Cell culture section. Cells were fixed with 4% paraformaldehyde, permeabilized with 0.1% Triton X-100, covered with blocking solution (3% BSA/PBS), and then stained with Alexa 594-conjugated phalloidin (A12381, Thermo Fisher Scientific). In some experiments, cells were incubated with an anti-Ki-67 primary antibody followed by an Alexa 488-conjugated anti-rabbit IgG secondary antibody (A21206, Thermo Fisher Scientific). Coverslips were mounted using Vectashield Hard Set with DAPI (Vector Laboratories, Newark, CA). Each specimen was visualized under the All-in-One Fluorescence Microscope BZ-9000 (KEYENCE, Osaka, Japan). In some experiments, the percentage of positive immunolabeled cells was counted in ten selected areas in a masked manner.

### Mice and xenografts

S2-013 cell line-derived organoids were established using cultured S2-013 cells, HUVECs, and MSCs as previously reported [21]. Animal experiments were approved by Kochi University (#R01-027), and mice were treated in accordance with the Institutional Animal Care and Use Committee guidelines of Kochi University. To generate subcutaneous tumors, a S2-013 cell line-derived organoid in 50 μL Matrigel matrix/DMEM mixture was subcutaneously implanted into the flanks of 7-week-old female athymic nude mice (BALB/cSlc-*nu/nu*) (Japan SLC, Inc., Shizuoka, Japan). After implantation, tumors were allowed to grow for 2 weeks before the initiation of treatment, as described below.

### Treatment regimens

Tumors were allowed to grow for 2 weeks after the implantation of S2-013 cell line-derived organoids into mice, and treatment was then initiated with (a) 8 mg/kg/day rapamycin by an intraperitoneal injection (ip) 5 days per week for 6 weeks (*n* = 8); (b) 50 mg/kg gemcitabine plus 0.5 mg/kg paclitaxel on days 1, 8, and 15 every 28 days for 6 weeks (*n* = 8) [23]; (c) the ip combination of 8 mg/kg/day rapamycin 5 days per week with 50 mg/kg gemcitabine plus 0.5 mg/kg paclitaxel on days 1, 8, and 15 every 28 days for 6 weeks (*n* = 8); (d) 2 mg/kg everolimus administered orally 5 days per week for 6 weeks (*n* = 8) [22]; and (e) the combination of 2 mg/kg everolimus administered orally 5 days per week with 50 mg/kg gemcitabine plus 0.5 mg/kg paclitaxel ip on days 1, 8, and 15 every 28 days for 6 weeks (*n* = 8). In addition, tumors were allowed to grow for 2 weeks after the implantation of S2-013 cell line-derived organoids into mice, and treatment was then initiated with (a) S-1 administered orally 5 days per week for 28 days with a rest period of 2 weeks as one course for 8 weeks (*n* = 8) [23]; (b) the combination of 1.5 mg/kg/day rapamycin ip 5 days per week with S-1 administered orally 5 days per week for 28 days with a rest period of 2 weeks as one course for 6 weeks (*n* = 8); and (c) the combination of 2 mg/kg everolimus administered orally 5 days per week with S-1 administered orally 5 days per week for 28 days with a rest period of 2 weeks as one course for 6 weeks (*n* = 8). Tumor sizes were measured every week, and tumor volumes were calculated with the following formula: V (mm^3^) = 0.5 × a × b^2^, where a and b represent the long and perpendicular short diameters of tumors, respectively. Mice were sacrificed eight weeks after the implantation of S2-013 cell line-derived organoids, and solid tumors, the lungs, liver, and kidneys were dissected. Solid tumor, lung, and liver sections were stained with hematoxylin and eosin.

### Immunohistochemical staining for Ki-67 and terminal deoxynucleotidyl transferase-mediated nick end labeling (TUNEL)

A proliferation analysis was performed on paraffin-embedded xenograft tumor tissues obtained from xenografts of S2-013 cell-line organoid-bearing mice by immunohistochemistry using the anti-Ki-67 antibody [15]. S2-013 cell line-derived organoids were implanted subcutaneously into the flanks of female athymic nude mice, as described in the Mice and xenografts section, followed by treatments described in the Treatment regimens section. Tissue sections from formalin-fixed paraffin-embedded xenograft tumors were stained with the anti-Ki-67 antibody, and the DAKO EnVision System (Glostrup, Denmark), containing a secondary horseradish peroxidase-conjugated anti-mouse antibody, was used with 3,3'-diaminobenzidine to detect Ki-67. TUNEL was performed on tissue sections from xenograft tumors of S2-013 cell-line organoid-bearing mice treated with the treatment regimens described above using the In Situ Cell Death Detection Kit (Roche Diagnostics, Basel, Switzerland), according to the manufacturer’s instructions. A positive reaction was defined as the intracellular distribution of brown coarse particles or the diffuse distribution of brownish yellow fine particles. Stained cells were counted in 10 random microscopic fields under a light microscope.

### Statistical analysis

StatFlex software (Ver6; YUMIT, Osaka, Japan) and SAS software (Ver9.1.3; SAS Institute, Cary, NC) were used for statistical analyses. The significance of differences between groups was assessed using the two-tailed Student’s *t*-test or Fisher’s exact test, as appropriate. In all analyses, *p* < 0.05 was considered to be significant.

## Results

### *Effects of rapamycin on apoptosis and the inhibition of cell proliferation *in vitro

**Fig. 1:**
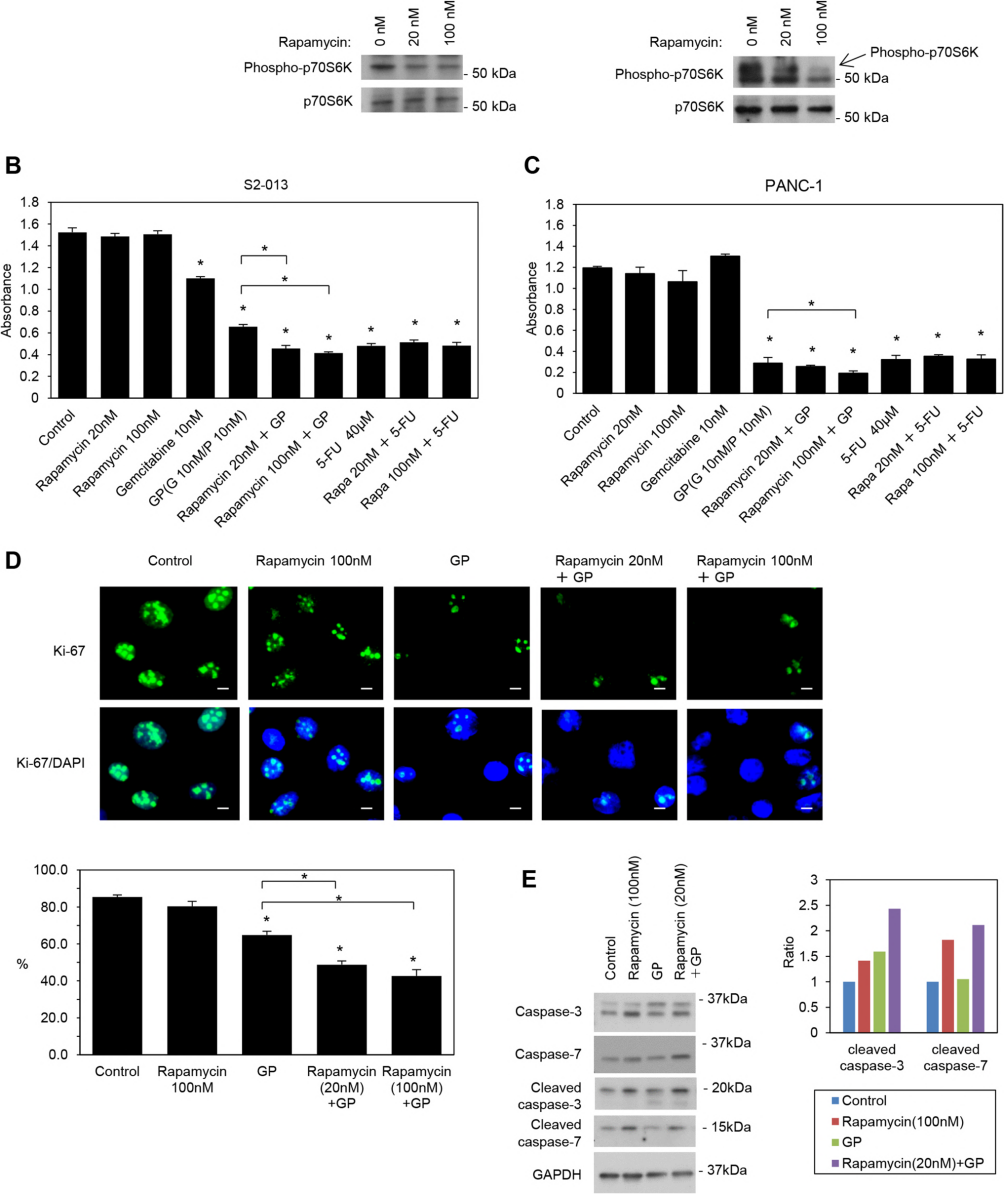
Effects of rapamycin on apoptosis and the inhibition of cell proliferation in vitro. **A** Western blotting was performed on S2-013 and PANC-1 cells treated with rapamycin using an anti-phosphorylated p70S6K antibody. **B**, **C** The MTT assay was performed on S2-013 (**B**) and PANC-1 (**C**) cells treated with rapamycin, gemcitabine, GP, rapamycin plus GP, 5-FU, and rapamycin plus 5-FU. The concentrations of rapamycin were 20 and 100 nM, that of gemcitabine was 10 nM, that of paclitaxel was 10 nM, and that of 5-FU was 40 μM. Data were derived from three independent experiments. *Columns*, mean; *bars*, SD. *p < 0.05 significantly different from non-treated control cells (the Student’s *t*-test). **D** Confocal immunofluorescence microscopic images of Ki-67 (green) in S2-013 cells treated with rapamycin, GP, and rapamycin plus GP. Blue, DAPI staining. Scale bars, 10 μm. Quantification of data; values represent the number of cells stained with the anti-Ki-67 antibody. All cells in four visual fields per group were scored. Data were derived from three independent experiments. Columns, mean; bars, SD. *p < 0.05 significantly different from non-treated control cells (Fisher’s exact test). **E** Western blotting was performed on S2-013 cells treated with rapamycin, GP, and rapamycin plus GP using anti-cleaved caspase-3 and anti-cleaved caspase-7 antibodies (left panel). Rapamycin was used at a concentration of 100 nM, while gemcitabine and paclitaxel were both used at 10 nM. When combined, all drugs, except rapamycin, were used at the same concentration (10 nM), with rapamycin being used at a lower concentration of 20 nM. A baseline value was calculated by subtracting the blank value from the measured GAPDH value in the group (control, rapamycin, GP, and rapamycin plus GP) (right panel). The value for each antibody (anti-cleaved caspase-3 and anti-cleaved caspase-7 antibodies) was then calculated by subtracting the blank value from the corresponding measured value. The ratio to the baseline was assessed by dividing the antibody value by the baseline value for the group. The combination of rapamycin with GP increased the ratio of cleaved caspase-3 and −7, indicating that this combination induced apoptosis

We compared the effects of rapamycin on apoptosis and the inhibition of cell proliferation in PDAC cells with those of current standard anti-PDAC drugs, including gemcitabine, paclitaxel (used as a substitute for nab-paclitaxel), and 5-FU. We used two types of cultured PDAC cell lines: moderately differentiated (S2-013) [27] and poorly differentiated (PANC-1) [28]. Immunoblotting showed that two concentrations of rapamycin, 20 and 100 nM, inhibited the activity of p70S6K, a downstream effector of mTOR, in a concentration-dependent manner in S2-013 and PANC-1 cells (Fig. 1A). Gemcitabine and GP significantly inhibited the viability of S2-013 cells (Fig. 1B), while GP significantly inhibited the viability of PANC-1 cells (Fig. 1C). Rapamycin increased the effects of GP in a concentration-dependent manner in S2-013 and PANC-1 cells (Fig. 1B and C). Rapamycin at all concentrations did not enhance the significant inhibitory effects of 5-FU on S2-013 and PANC-1 cell growth (Fig. 1B and C).

We then investigated whether the expression levels of Ki-67, a nuclear protein associated with cell proliferation, were affected in S2-013 cells treated with rapamycin, GP, or the combination of rapamycin with GP (Fig. 1D). Ki-67 expression levels did not significantly differ between cells treated with 100 nM rapamycin and non-treated control cells, while rapamycin decreased its expression levels in S2-013 cells treated with GP in a rapamycin concentration-dependent manner, similar to the results of MTT assays.

To examine the effects of treatments with rapamycin, GP, and the combination of rapamycin with GP on caspase cleavage in S2-013 cells, total cell lysates were prepared for Western blotting (Fig. 1E). The expression levels of the 19-kDa fragment of caspase-3 and the 15-kDa fragment of caspase-7 increased in S2-013 cells treated with 100 nM rapamycin and the combination of 20 nM rapamycin with GP. GP did not significantly change the expression levels of the 19-kDa fragment of caspase-3 or the 15-kDa fragment of caspase-7 from those in non-treated control cells (Fig. 1E). Even a low concentration of 20 nM rapamycin appeared to induce apoptosis when combined with GP.

### *Inhibitory effects of rapamycin on cell invasion *in vitro

**Fig. 2:**
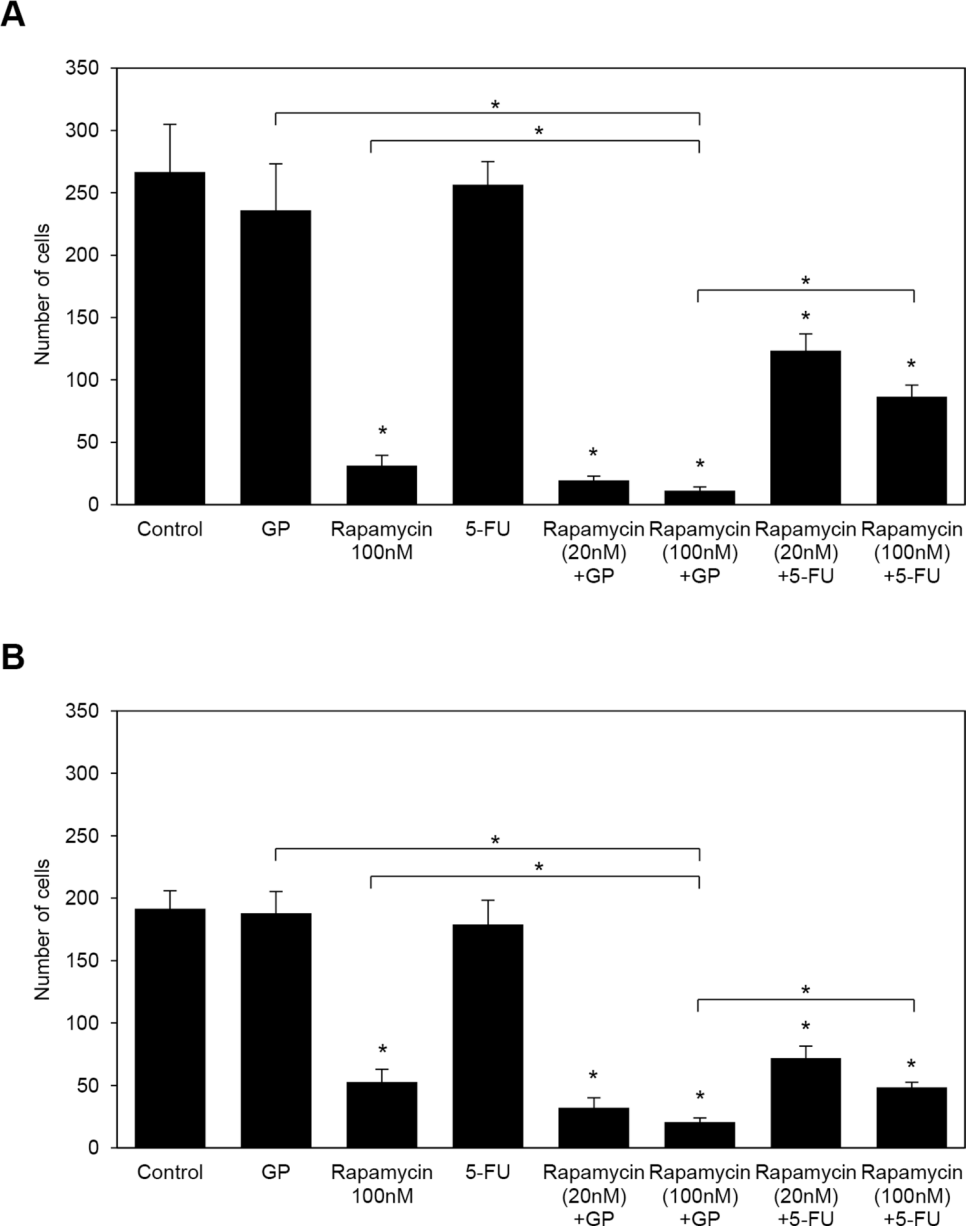
Effects of rapamycin on the inhibition of cell invasion in vitro. **A**, **B** S2-013 (**A**) and PANC-1 (**B**) cells were incubated with rapamycin, GP, rapamycin plus GP, 5-FU, and rapamycin plus 5-FU. The concentration of rapamycin was 100 nM, that of gemcitabine was 10 nM, that of paclitaxel was 10 nM, and that of 5-FU was 40 μM. When combined, all drugs, except for rapamycin, were used at the same concentration (10 nM), with rapamycin being used at 20 and 100 nM. Two-chamber invasion assays were performed, and migrating cells in four fields per group were then scored. Data were derived from three independent experiments. Columns, mean; bars, SD. *p < 0.05 significantly different from non-treated control cells (the Student’s *t*-test)

To establish whether a rapamycin treatment combined with or without GP or 5-FU affected the invasiveness of PDAC cells, in vitro invasion assays were performed. The inhibition of cell invasion was significantly stronger in cells treated with 100 nM rapamycin than in non-treated control cells, and GP and 5-FU did not affect invasiveness in S2-013 or PANC-1 cells (Fig. 2A for S2-013 and Fig. 2B for PANC-1). It is important to note that the combination of rapamycin with GP and rapamycin plus 5-FU significantly increased their inhibitory effects on cell invasion in a rapamycin concentration-dependent manner in S2-013 and PANC-1 cells (Fig. 2A and B). Among these treatments, the combination of rapamycin with GP was the strongest inhibitor of the invasion of S2-013 and PANC-1 cells.

### Inhibitory effects of rapamycin on the formation of cell protrusions in PDAC cells

**Fig. 3:**
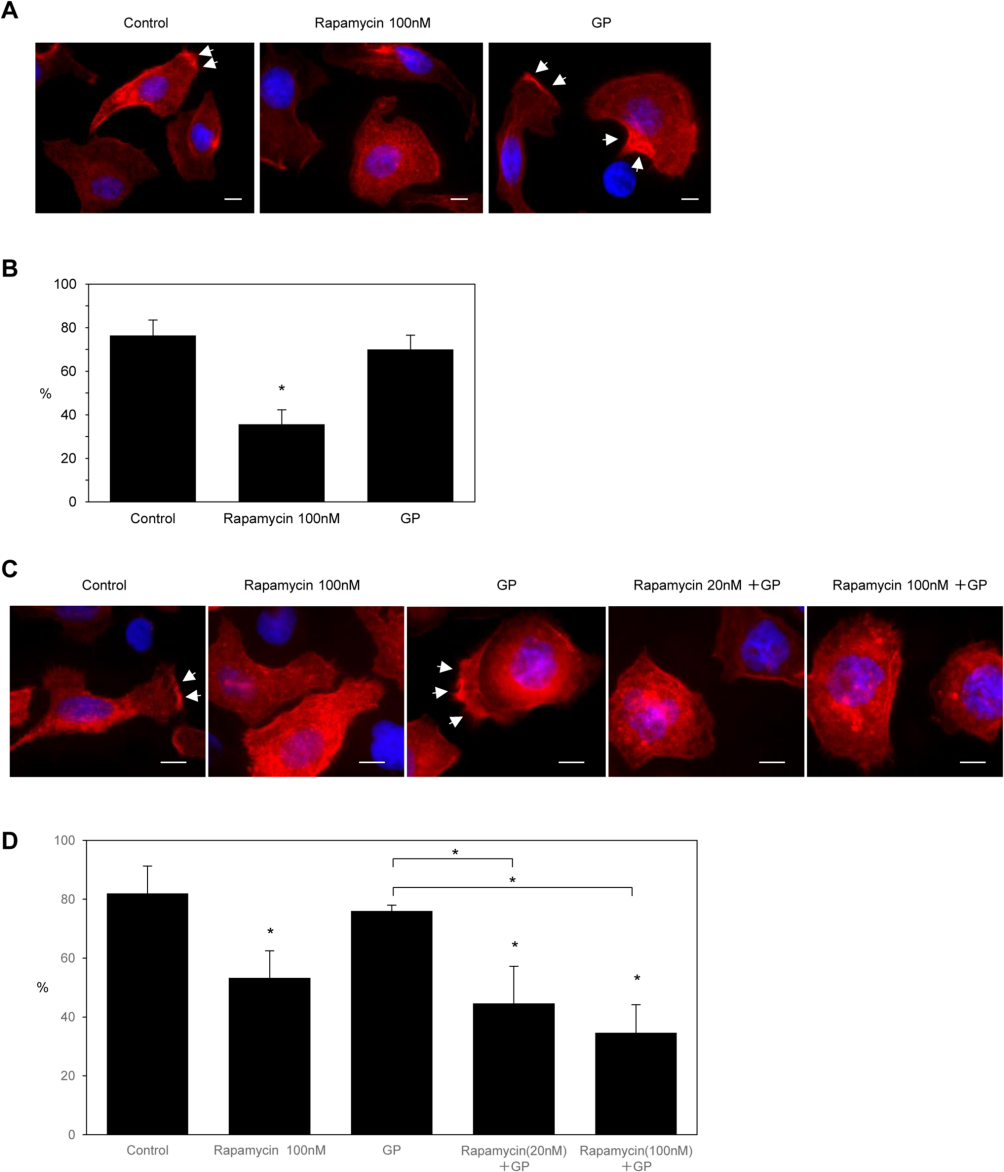
Inhibitory effects of rapamycin on the formation of cell protrusions in PDAC cells. **A** Confocal immunofluorescence microscopic images. S2-013 cells were incubated with rapamycin and GP. The concentration of rapamycin was 100 nM, that of gemcitabine was 10 nM, and that of paclitaxel was 10 nM. Cells were stained with phalloidin (red). Blue, DAPI staining. Arrows, peripheral actin structures in cell protrusions. Bars, 10 µm. **B** Quantification of data shown in Fig. 3A; values represent the number of cells with cell protrusions in which peripheral actin structures increased. All cells in four fields per group were scored. Data were derived from three independent experiments. Columns, mean; bars, SD. *p < 0.05 significantly different from non-treated control cells (Fisher’s exact test). **C** Confocal immunofluorescence microscopic images. S2-013 cells were incubated with rapamycin, GP, and rapamycin plus GP. Rapamycin was used at a concentration of 100 nM, while gemcitabine and paclitaxel were both used at 10 nM. When combined, all drugs, except for rapamycin, were used at the same concentration (10 nM), with rapamycin being used at 20 and 100 nM. Cells were stained with phalloidin (red). Arrows, cell protrusions. Blue, DAPI staining. Bars, 10 µm. **D** Quantification of data shown in Fig. 3C; values represent the number of cells with cell protrusions in which peripheral actin structures increased. All cells in four fields per group were scored. Data were derived from three independent experiments. Columns, mean; bars, SD. *p < 0.05 significantly different from non-treated control cells (Fisher’s exact test)

To investigate whether the treatment with rapamycin inhibited the formation of membrane protrusions, we examined peripheral actin structures in the membrane ruffles of S2-013 cells. Immunocytochemistry showed that there were fewer peripheral actin structures in cells treated with 100 nM rapamycin than in non-treated control cells (Fig. 3A). Furthermore, the formation of membrane protrusions was significantly lower in cells treated with rapamycin than in non-treated control cells (Fig. 3B). Conversely, the inhibition of surface actin rearrangements and the formation of cell protrusions were not observed in S2-013 cells treated with GP (Fig. 3B). We then examined the actin cytoskeletal structures of S2-013 cells treated with rapamycin in the absence or presence of GP. Immunocytochemistry showed that the addition of rapamycin to GP increased the inhibition efficacy of rapamycin on the surface actin rearrangement (Fig. 3C) and the formation of cell protrusions (Fig. 3D) over that of GP in a rapamycin concentration-dependent manner in S2-013 cells. These results indicated that rapamycin plays a role in inhibiting peripheral actin-cytoskeletal rearrangements and also that the combination of rapamycin with GP was the strongest inhibitor of the formation of cell protrusions in S2-013 cells.

### *Effects of everolimus on apoptosis, cell proliferation, and invasion *in vitro

**Fig. 4:**
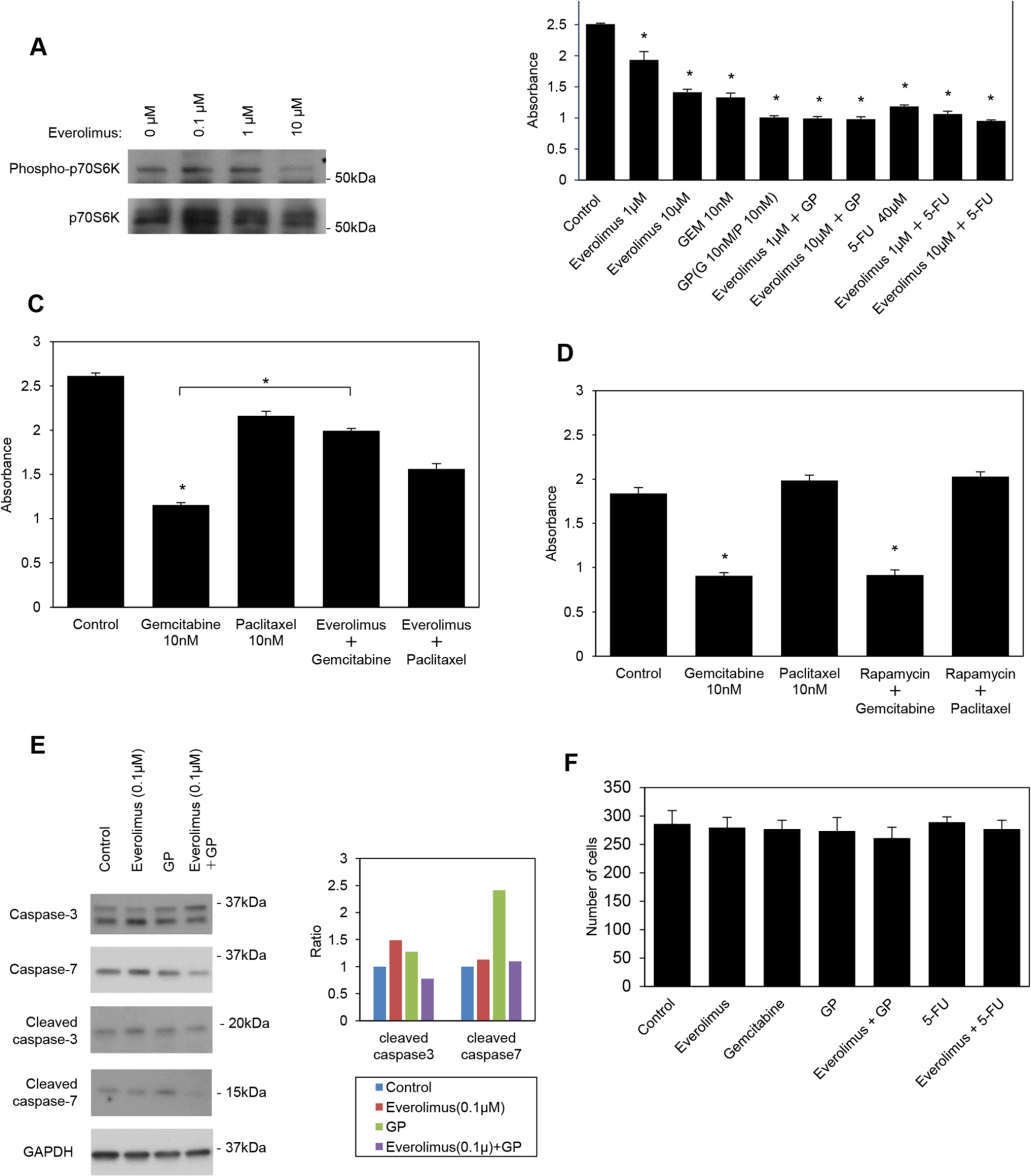
Effects of everolimus on apoptosis, cell proliferation, and invasion in vitro. **A** Western blotting was performed on S2-013 cells treated with everolimus using the anti-phosphorylated p70S6K antibody. **B** The MTT assay was performed on S2-013 cells treated with everolimus, gemcitabine, GP, everolimus plus GP, 5-FU, and everolimus plus 5-FU. The concentrations of everolimus were 1 and 10 µM, that of gemcitabine was 10 nM, that of paclitaxel was 10 nM, and that of 5-FU was 40 μM. Data were derived from three independent experiments. Columns, mean; bars, SD. *p < 0.05 significantly different from non-treated control cells (the Student’s *t*-test). **C** The MTT assay was performed on S2-013 cells treated with gemcitabine, everolimus plus gemcitabine, paclitaxel, and everolimus plus paclitaxel. The concentration of everolimus was 10 µM, that of gemcitabine was 10 nM, and that of paclitaxel was 10 nM. Data were derived from three independent experiments. Columns, mean; bars, SD. *p < 0.05 significantly different from non-treated control cells (the Student’s *t*-test). **D** The MTT assay was performed on S2-013 cells treated with gemcitabine, rapamycin plus gemcitabine, paclitaxel, and rapamycin plus paclitaxel. The concentration of rapamycin was 100 nM, that of gemcitabine was 10 nM, and that of paclitaxel was 10 nM. Data were derived from three independent experiments. Columns, mean; bars, SD. *p < 0.05 significantly different from non-treated control cells (the Student’s *t*-test). **E** Western blotting was performed on S2-013 cells treated with everolimus, GP, and everolimus plus GP using anti-cleaved caspase-3 and anti-cleaved caspase-7 antibodies (left panel). The concentration of everolimus was 1 µM, that of gemcitabine was 10 nM, and that of paclitaxel was 10 nM, A baseline value was calculated by subtracting the blank value from the measured GAPDH value in the group (control, everolimus, GP, and everolimus plus GP) (right panel). The value for each antibody (anti-cleaved caspase-3 and anti-cleaved caspase-7 antibodies) was then calculated by subtracting the blank value from the corresponding measured value. The ratio to the baseline was assessed by dividing the antibody value by the baseline value for the group. The combination of everolimus with GP did not increase these apoptotic markers. **F** S2-013 cells were incubated with everolimus, gemcitabine, GP, everolimus plus GP, 5-FU, and everolimus plus 5-FU. The concentration of everolimus was 10 µM, that of gemcitabine was 10 nM, that of paclitaxel was 10 nM, and that of 5-FU was 40 μM. Two-chamber invasion assays were performed, and migrating cells in four fields per group were then scored. Data were derived from three independent experiments. Columns, mean; bars, SD

To establish whether a treatment with another mTOR inhibitor, everolimus was associated with apoptosis, cell proliferation, and invasion, S2-013 cells were treated with everolimus at concentrations of 0.1, 1, and 10 μM. Immunoblotting showed that p70S6K activity was significantly lower in S2-013 cells treated with 10 μM everolimus than in those treated with 0.1 and 1 μM (Fig. 4A). In contrast to rapamycin, everolimus significantly inhibited the viability of S2-013 cells in a concentration-dependent manner (Fig. 4B), and the inhibitory effects of 10 μM everolimus on cell proliferation were similar to those of gemcitabine and 5-FU. Although rapamycin enhanced the inhibitory effects of GP on cell proliferation in a concentration-dependent manner, as shown in Fig. 1B, the addition of everolimus did not potentiate the inhibitory effects of GP on the proliferation of S2-013 cells (Fig. 4B). When the combination of everolimus and gemcitabine was evaluated, everolimus inhibited the antiproliferative activity of gemcitabine in S2-013 cells (Fig. 4C). The inhibitory effects of gemcitabine on cell proliferation were not affected by rapamycin in S2-013 cells (Fig. 4D). Similar to rapamycin, the presence of everolimus did not affect the ability of 5-FU to inhibit the proliferation of S2-013 cells (Fig. 4B). It is important to note that everolimus significantly differed from rapamycin in that it was involved in the inhibition of cell proliferation, but did not exert synergistic effects with GP, in contrast to rapamycin.

Regarding the effects of everolimus-based treatments on caspase cleavage, immunoblotting showed that the expression levels of the 19-kDa fragment of caspase-3 and the 15-kDa fragment of caspase-7 did not significantly differ between cells treated with everolimus and the combination of everolimus and GP and non-treated control cells, in contrast to rapamycin (Fig. 4E).

To clarify whether the treatment with everolimus in combination with or without GP or 5-FU affected the invasiveness of PDAC cells, in vitro invasion assays were performed. In contrast to rapamycin, everolimus failed to inhibit cell invasion, similar to that in non-treated control cells, and the treatment with GP and that with 5-FU did not affect the invasiveness of S2-013 cells (Fig. 4E). Furthermore, in contrast to rapamycin, the combination of everolimus with GP and the combination of everolimus with 5-FU did not exert additive effects with GP or 5-FU in terms of the inhibition of invasion by S2-013 cells (Fig. 4F).

### Inhibitory effects of rapamycin-based treatments on the tumor growth of xenografts in the S2-013-organoid model

**Fig. 5:**
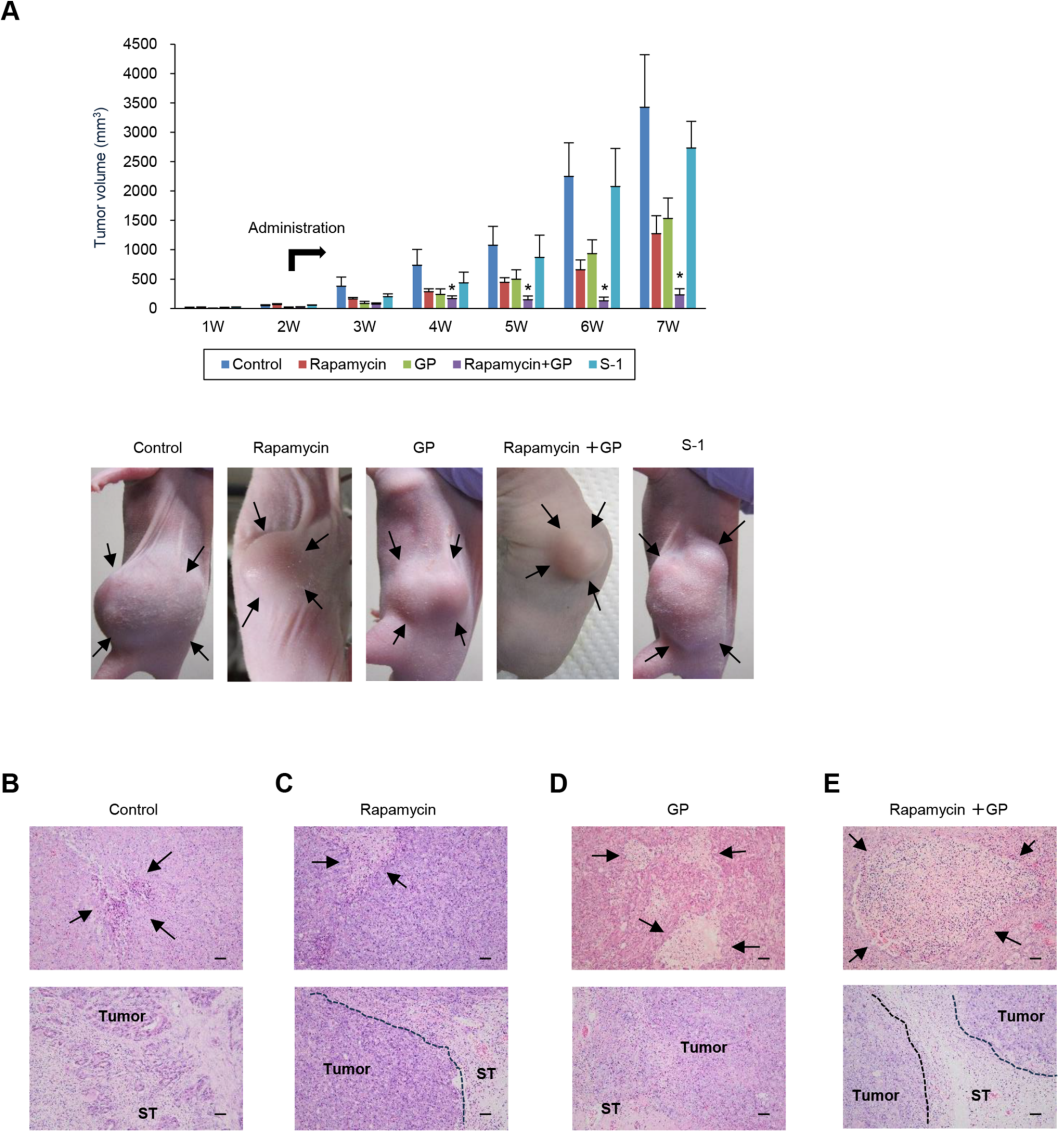
Inhibitory effects of rapamycin-based treatments on tumor growth in xenografts of the S2-013-organoid model. **A** Two weeks after the implantation of the S2-013-derived organoid, mice in the non-treated control, rapamycin, GP, rapamycin plus GP, and S-1 groups (*n* = 8 each) received the first treatment, and all mice received each treatment for six weeks. Average tumor volumes and representative images of mice in each treatment group were obtained one week after the completion of six weeks of administration. The images show the gross appearance of mice. Columns, mean; bars, SEM. *p < 0.05 significantly different from the rapamycin-treated group and GP-treated group (Fisher’s exact test). Tumor volumes were compared between the groups on a weekly basis. Arrows, xenografts. **B**–**E** Representative HE staining of the tumor xenograft obtained from mice in each group described in Fig. 5A eight weeks after transplantation. Tumor necrosis (upper panel, arrows) and peritumoral subcutaneous infiltration (lower panel) in the non-treated control group (**B**), tumor necrosis (upper panel, arrows) and the inhibition of peritumoral subcutaneous infiltration (lower panel) in the rapamycin-treated group (**C**), tumor necrosis (upper panel, arrows) and peritumoral subcutaneous infiltration (lower panel) in the GP-treated group (**D**), and tumor necrosis (upper panels, arrows) and the inhibition of peritumoral subcutaneous infiltration (lower panel) in the rapamycin plus GP-treated group (**E**). Dotted line: tumor-infiltrating margin. ST, subcutaneous tissue. Bars, 50 µm

To examine the effects of rapamycin-based treatments on cancer cell dynamics in vivo, the nude mouse model of PDAC established by the subcutaneous implantation of S2-013-derived organoids into the dorsal regions of mice (S2-013-organoid model) was used [21]. Nab-paclitaxel is produced by the combination of paclitaxel and human albumin. We used paclitaxel instead of nab-paclitaxel due to concerns that mice may have allergic reactions to human albumin in this mouse experiment. Furthermore, S-1, a metabolically activated prodrug of 5-FU, was used instead of 5-FU in the mouse experiment. S-1 has been approved in adjuvant chemotherapy for resected PDAC in Japan [29]. Two weeks after the implantation of the S2-013-derived organoid, mice in the non-treated control, rapamycin, GP, combination of rapamycin with GP, and S-1 groups received the first treatment, as described in the Materials and methods section. Comparisons of photomicrographs of tumors one week after the completion of 6 weeks of treatment with those in non-treated controls showed that GP significantly suppressed overall tumor growth (Fig. 5A). Tumor suppression was significantly greater in the S-1 group than in the control group; however, its efficacy was inferior to that of GP (Fig. 5A). The efficacy of rapamycin to suppress tumor growth was slightly superior to that of GP, and the combination of rapamycin with GP suppressed tumor growth significantly more than GP or rapamycin alone (Fig. 5A). Notably, rapamycin exerted an additive effect on the inhibition of tumor growth by GP.

Eight weeks after implantation, mice were sacrificed, xenograft sections were prepared, and hematoxylin and eosin staining was performed to evaluate potential histopathological changes in the tissue architecture caused by rapamycin-based treatments (Fig. 5B–E). The necrotic area of the tumor was markedly larger after the treatment with GP than after that with rapamycin (Fig. 5C and D, upper panels). The addition of rapamycin to GP clearly increased the area of tumor necrosis induced by GP (Fig. 5D and E, upper panels). Adenocarcinoma with the regional invasion of subcutaneous tissue was observed in xenografts obtained from the control (Fig. 5B, lower panel), and infiltrative findings were noted in xenografts after the treatment with GP to the same extent as those in the control (Fig. 5B and D, lower panels). In contrast, the extent to which rapamycin prevented subcutaneous tissue infiltration was markedly lower than in the control (Fig. 5B and C, lower panels). The combination of rapamycin with GP resulted in two types of pathological findings: one with suppressed subcutaneous tissue infiltration to the same extent as in xenografts after the treatment with rapamycin and the other with multiple necrotic areas to the same extent as in GP xenografts (Fig. 5E). Therefore, the combination of rapamycin with GP appeared to have suppressed tumor growth via two mechanisms: the induction of necrosis in tumor cells as a therapeutic effect of GP and the inhibition of invasion into surrounding subcutaneous tissues as a therapeutic effect of rapamycin.

### Inhibitory effects of everolimus-based treatments on the growth of xenograft tumors in S2-013 cell-line organoid-bearing mice

**Fig. 6:**
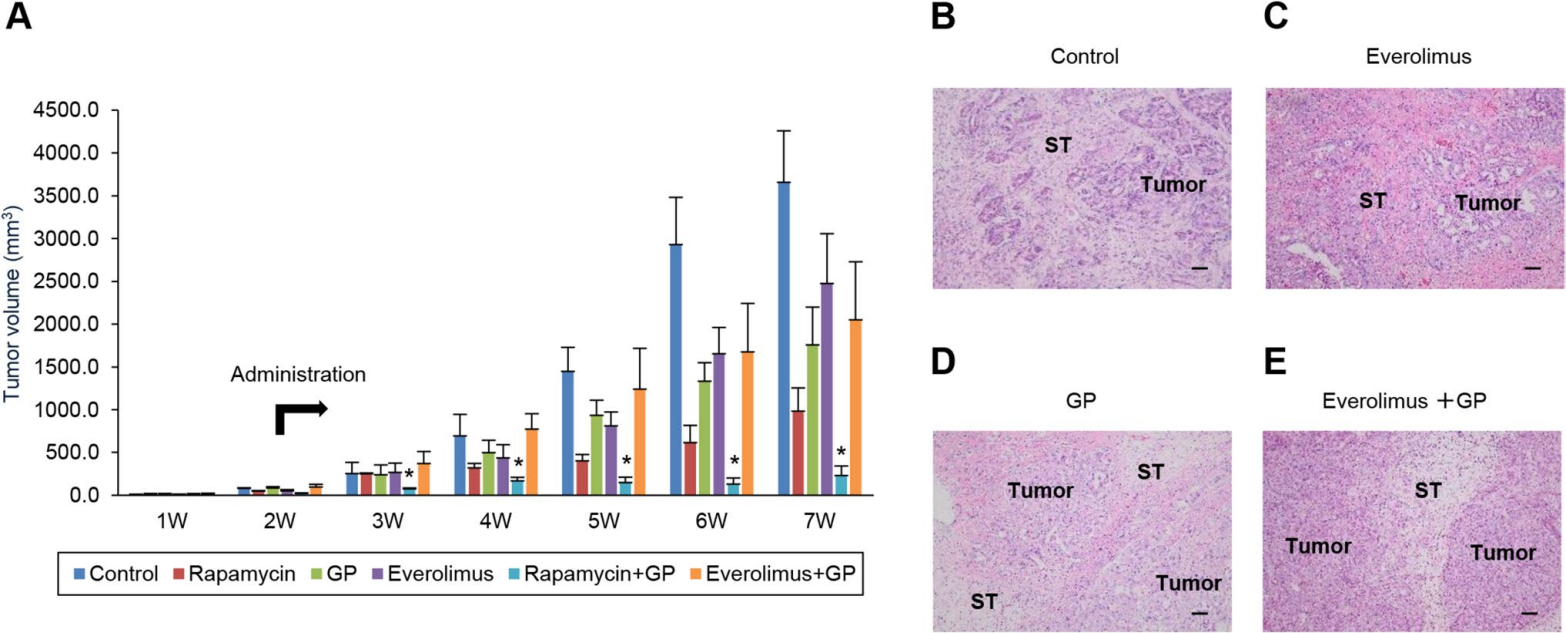
Inhibitory effects of everolimus-based treatments on tumor growth in xenografts of the S2-013-organoid model. **A** Two weeks after the implantation of the S2-013-derived organoid, mice in each group (*n* = 8) of the non-treated control, rapamycin, everolimus, GP, everolimus plus GP, and rapamycin plus GP received the first treatment, and all mice received each treatment for six weeks. Average tumor volumes and representatives from each treatment group are shown. Columns, mean; bars, SEM. *p < 0.05 significantly different from the rapamycin-treated group, GP-treated group and everolimus plus GP-treated group (Fisher’s exact test). Tumor volumes were compared between the groups on a weekly basis. **B**–**E** Representative HE staining of tumor xenografts obtained from mice in each group described in Fig. 6A eight weeks after transplantation. Peritumoral subcutaneous infiltration in the non-treated control group (**B**), everolimus-treated group (**C**), GP-treated group (**D**), and everolimus plus GP-treated group (**E**). ST, subcutaneous tissue. Bars, 50 µm

Two weeks after the implantation of the S2-013-derived organoid, mice in the non-treated control, rapamycin, everolimus, GP, combination of rapamycin with GP, and combination of everolimus with GP groups received the first treatment, as described in the Materials and methods section. The combination of rapamycin with GP significantly enhanced the tumor shrinkage effects of rapamycin alone and GP, whereas the combination of everolimus with GP was inferior to rapamycin alone and GP in terms of tumor shrinkage in the S2-013-organoid model (Fig. 6A). Eight weeks after implantation, mice were sacrificed, and xenograft sections were prepared. Hematoxylin and eosin staining showed no significant changes in the subcutaneous invasion of PDAC cells among the control, everolimus, GP, and combination of everolimus with GP groups (Fig. 6B–E).

### Comparative effects of rapamycin and everolimus on xenograft residual cell proliferation and apoptosis

**Fig. 7:**
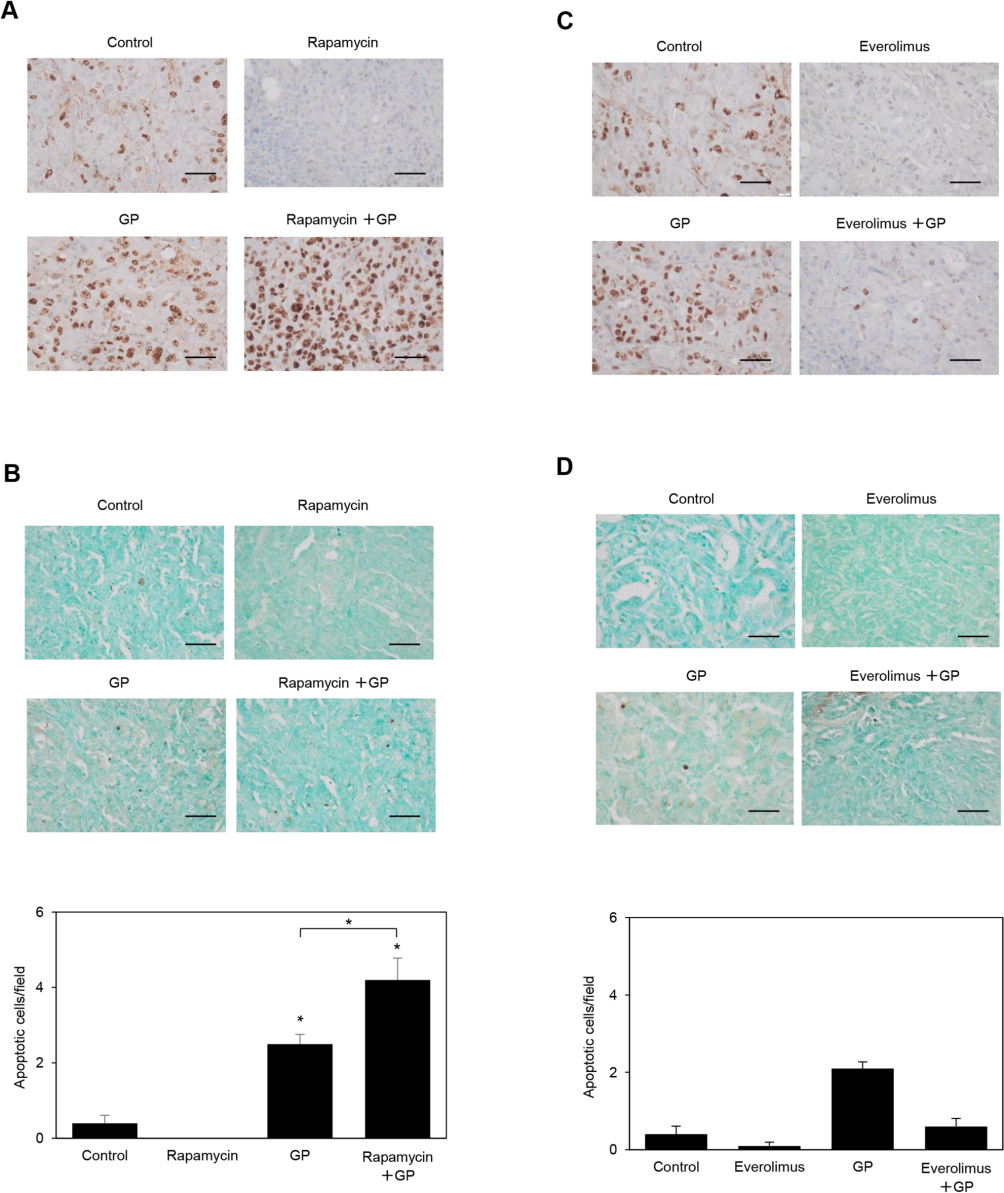
Post-treatment effects of rapamycin and everolimus on tumor cell proliferation and apoptosis. **A** Immunohistochemical staining with the anti-Ki-67 antibody of xenografts obtained from S2-013-organoid mice after the completion of treatment with rapamycin, GP, and rapamycin plus GP. Bars, 50 µm. **B** TUNEL assay on xenografts obtained from S2-013-organoid mice after the completion of treatment with rapamycin, GP, and rapamycin plus GP. Values represent the number of TUNEL-positive cells. All cells in four fields per group were scored. Data were derived from three independent experiments. Columns, mean; bars, SEM. *p < 0.05 significantly different from non-treated control cells (the Student’s *t*-test). Bars, 50 µm. **C** Immunohistochemical staining with the anti-Ki-67 antibody in xenografts obtained from S2-013-organoid mice after the completion of treatment with everolimus, GP, and everolimus plus GP. Bars, 50 µm. **D** TUNEL assay on xenografts obtained from S2-013-organoid mice after the completion of treatment with everolimus, GP, and everolimus plus GP. Values represent the number of TUNEL-positive cells. All cells in four fields per group were scored. Data were derived from three independent experiments. Columns, mean; bars, SEM. Bars, 50 µm

Regarding rapamycin-based treatment-resistant residual cells within xenografts, representative assessments of the proliferation and apoptosis of tumor cells with or without treatment are shown in Fig. 7. The immunohistochemical analysis of xenograft sections obtained eight weeks after implantation showed a marked increase in the population of Ki-67-positive cells following treatment with GP (Fig. 7A). Ki-67-positive cells were decreased by the treatment with rapamycin, whereas the addition of rapamycin to GP resulted in synergistic effects, leading to the even greater expansion of the positive cell population (Fig. 7A). The TUNEL assay revealed a significantly higher number of apoptotic cells after the treatment with GP and the combination of rapamycin with GP than that with the control or rapamycin treatment alone (Fig. 7B). Rapamycin decreased both the number of Ki-67-positive cells and apoptotic cells in the remaining cancer cells. Interestingly, it potentiated the effects of GP, which increased the number of Ki-67-positive cells, but also induced apoptosis in the residual cells.

The immunohistochemical analysis of xenograft sections obtained eight weeks after implantation showed that the number of Ki-67-positive cells decreased in the residual cancer cells of xenografts treated with everolimus; however, in contrast to rapamycin, the addition of everolimus to GP decreased the number of Ki-67-positive cells in the remaining cancer cells (Fig. 7C). Similar to rapamycin, the everolimus treatment resulted in fewer apoptotic cells detected by the TUNEL assay in the remaining cancer cells than in the control group (Fig. 7D). In contrast to rapamycin, everolimus decreased the number of apoptotic cells induced by GP (Fig. 7D). The key difference between rapamycin and everolimus lies in their opposing effects on the GP-mediated proliferation and apoptosis of residual cancer cells. Rapamycin potentiated these effects, whereas everolimus suppressed them. A comparison of the effects of the combination of rapamycin with GP and the combination of everolimus with GP on the inhibition of proliferation, invasion, and apoptosis and on tumor volumes is summarized in Table 1.

**Table 1:** Differential effects of rapamycin and everolimus, combined with GP, on cell proliferation, apoptosis, and cell invasion

	Rapamycin plus GP	Everolimus plus GP
Proliferation in vitro	Stronger inhibitory effect than GP	Same inhibitory effect as GP
Invasion in vitro	Strong inhibitory effect (GP has no inhibitory effect.)	No inhibitory effect similar to GP
Apoptosis in vitro	Stronger inducing effect than GP	No inducing effect (GP induces apoptosis.)
Tumor growth in vivo	Stronger suppressive effect than GP	Same suppressive effect as GP
Proliferation in residual cells in vivo Apoptosis in residual cells in vivo	Stronger inducing effect than GP Stronger inducing effect than GP	Strong inhibitory effect (GP induces proliferation.) Strong inhibitory effect (GP induces apoptosis.)

## Discussion

Despite global research and development efforts, the approval of new drugs as first-line chemotherapy for advanced PDAC has been stagnant for an extended period. While clinical trials on molecular targeted agents and immune checkpoint inhibitors are ongoing for PDAC, their therapeutic efficacy remains limited [30, 31]. A cancer genome analysis revealed frequent abnormalities in four genes: *KRAS*, *CDKN2A*/*p16*, *TP53*, and *SMAD4*/*DPC4* [32–35]. Mutations in these genes occur at different stages of precursor lesions, and their dysregulation promotes the differentiation and proliferation of PDAC [36]. Among the development of molecular targeted drugs against *KRAS*, *CDKN2A*/*p16*, *TP53*, and *SMAD4*/*DPC4*, only *KRAS*^G12C^ inhibitors have achieved success. Although a phase I/II trial on the *KRAS*^G12C^ inhibitor Lumakras (sotorasib), which was approved for unresectable non-small cell lung cancer by the United States Food and Drug Administration in 2021, has demonstrated efficacy for *KRAS*^G12C^-mutated advanced PDAC patients who received previous treatment [37], the findings obtained need to be verified in a phase III clinical trial prior to its approval as a treatment for *KRAS*^G12C^-mutated advanced PDAC. *KRAS*^G12C^ mutations were identified in approximately 1.0% of PDAC patients (29 of 3,051) [38]. With a prevalence of only 1.0% in PDAC, *KRAS*^G12C^ mutations may leave most patients without an approved treatment option, even if an inhibitor is approved.

The Japan Pancreatic Cancer Treatment Guidelines 2022 recommend GnP and FOLFIRINOX as first-line chemotherapy for advanced PDAC that is not surgically resectable [39]. Indirect and retrospective analyses suggested the superior survival benefits of FOLFIRINOX over GnP, whereas an intermediate analysis of a phase II/III study (the JCOG1611-GENERATE trial), a direct comparison of the two treatments in patients with metastatic or recurrent PDAC, showed that GnP (median OS: 17.1 months) was not inferior to FOLFIRINOX (median OS: 14.0 months) in the final analysis, and GnP was recommended as first-line chemotherapy for patients with metastatic or recurrent PDAC [40, 41]. The incidence of grade 3 or higher non-hematological treatment-related adverse events was 5.2% in the GnP group and 22.8% in the FOLFIRINOX group, and GnP was also superior in terms of safety.

While GnP therapy and molecular targeted therapy exert their antitumor effects by inhibiting cell proliferation, neither possess anti-invasive properties. Since PDAC is characterized by its invasive nature and a poor prognosis [42], this is a critical limitation. Various cellular signals contribute to the regulation of invasion by PDAC cells. A comprehensive genetic analysis revealed a correlation between the expression levels of IL-32, PTX3, ARHGDIB, and PCYT1B and the invasiveness of PDAC [43]. Additionally, the aggressive nature of PDAC is driven by the dense desmoplastic stroma, which is rich in fibrous components [44]. The dense desmoplastic stroma creates a high-pressured barrier that collapses blood vessels and also inhibits the entry of immune cells and anticancer drugs into tumor tissue [45, 46]. Therefore, the development of new therapies that suppress “tumor invasion with desmoplasia”, a hallmark of PDAC and a major contributor to its poor prognosis, is urgently needed.

We investigated the mechanisms underlying PDAC invasion and metastasis, and identified mTOR as a key mediator of these processes [12]. Rapamycin (Sirolimus), an mTOR inhibitor, is currently approved in Japan for the treatment of lymphangiomatosis [47]. The recommended dosage for adults is 2 mg of rapamycin orally once daily. This dosage may be adjusted based on the patient’s condition, but cannot exceed 4 mg once daily. In the United States, rapamycin is approved for the prevention of graft rejection in kidney transplant patients aged 13 years and older in combination with cyclosporine. The initial dosage is 15 mg once daily, followed by a maintenance dosage of 5 mg once daily. It has not yet received regulatory approval for the treatment of malignant tumors. To evaluate the efficacy of rapamycin in PDAC, in vivo experiments were conducted in the present study. The results obtained showed that the inhibition of tumor growth was significantly stronger by the combination of rapamycin with GP than by GP or rapamycin alone through the suppression of tumor invasion into the subcutaneous space surrounding the tumor. Furthermore, no histological evidence of toxicity was detected in the lungs, liver, or kidneys of the S2-013-organoid model (n = 8) treated with the combination of rapamycin with GP for 10 weeks (data not shown). A phase II clinical trial (NCT00499486) evaluated the efficacy and safety of rapamycin monotherapy at 5 mg/day in 47 patients with unresectable, locally advanced, and metastatic PDAC that had progressed after first-line therapy [48]. There were no complete or partial responses. There were also no adverse events of interstitial pneumonitis, and grade 3 or higher adverse events only occurred in 9% of patients, which included hyperglycemia (1 patient), dehydration (3 patients), hypotension (1 patient), and asthenia (2 patients). Future clinical trials are needed to investigate whether the combination of rapamycin with GnP is superior to GnP monotherapy in terms of safety and response rates in patients with metastatic and recurrent PDAC.

Everolimus is used to treat various cancer types, such as unresectable renal cell carcinoma [49], digestive and pancreatic neuroendocrine tumors [50], unresectable breast cancer [51], and tuberous sclerosis complex [52]. It is a 4-hydroxy derivative of rapamycin with a slightly different structure [53]. Everolimus has a smaller molecular weight than rapamycin, making it more easily absorbed [53]. Additionally, everolimus does not associate with FKBP12, and instead inhibits mTORC1 and mTORC2 by directly blocking the ATP catalytic site, indicating that everolimus has higher selectivity for mTOR kinase than rapamycin [52, 54]. We investigated whether the slight structural and mTOR kinase selectivity differences between the two drugs led to changes in their antitumor efficacy against PDAC in the present study. While everolimus exhibited antiproliferative activity against PDAC cells, its effects were less potent than those of GP, but similar to those of gemcitabine in in vitro MTT assays. However, everolimus significantly reduced the antiproliferative activity of gemcitabine in MTT assays (Fig. 4C). Gemcitabine activates c-Jun N-terminal kinase and extracellular signal-regulated protein kinase (ERK), leading to the phosphorylation of Bcl-2 [55]. Everolimus reduced cell proliferation, and the phosphorylated ERK/ERK ratio in sinonasal intestinal-type adenocarcinoma showed significant decreases after 24 h of exposure to everolimus [56]. Moreover, everolimus was significantly more effective than rapamycin at inhibiting the mTOR signaling pathway, which may be mediated by the inhibition of ERK phosphorylation attenuating neuroinflammation in kainic acid-induced seizures [57]. These findings suggest that everolimus decreased the gemcitabine-mediated activation of ERK, thereby diminishing the antiproliferative activity of gemcitabine in MTT assays on S2-013 cells. Everolimus has been shown to inhibit cell proliferation in gemcitabine-treated PDAC cell lines in vitro [58], which is inconsistent with the present results; however, everolimus monotherapy did not exhibit antitumor activity in metastatic PDAC patients who experienced treatment failure on gemcitabine therapy [19]. Considering the combination of everolimus with GnP therapy in the same manner as rapamycin, there is a risk that everolimus may reduce the antiproliferative effects of gemcitabine.

The results obtained from in vitro experiments showed that rapamycin was not associated with the inhibition of cell proliferation or a decrease in the number of Ki-67-positive cells. However, when combined with GP, rapamycin inhibited cell proliferation and reduced the number of Ki-67-positive cells. In contrast, the results obtained from in vivo experiments demonstrated that rapamycin decreased the number of Ki-67-positive cells, while the combination of rapamycin with GP further enhanced the increase in Ki-67-positive cells induced by GP in residual cells after treatment. In vitro and in vivo experiments both demonstrated that rapamycin potentiated the effects of GP on cell proliferation and apoptosis. In vivo experiments specifically revealed that while rapamycin alone had no effect on apoptosis, combining it with GP further enhanced apoptosis induced by GP in residual cells after treatment.

It is important to note that everolimus did not exhibit anti-invasive activity against PDAC cells, unlike rapamycin in vitro. A histopathological examination of efficacy evaluation experiments using the S2-013-organoid model revealed that tumor growth was suppressed to a greater extent in the rapamycin plus GP group than in the everolimus plus GP or GP group. This enhanced suppression was attributed to the strong inhibition of invasion from the primary tumor to subcutaneous tissue in the rapamycin plus GP group, indicating that the suppression of tumor invasion is a crucial factor for achieving favorable response rates in PDAC. These results, which are summarized in Table 1, suggest that everolimus is not suitable for combination therapy with GP and lacks anti-invasive activity, which distinguishes it from rapamycin. Furthermore, immunohistochemical staining revealed that rapamycin increased the number of Ki-67-positive cells induced by GP in residual cells after treatment; however, the induction of apoptosis was only observed in combination with rapamycin. A previous study reported that the number of Ki-67-positive cancer cells in resected PDAC tissues was significantly higher in patients treated with neoadjuvant GnP therapy than in those treated with neoadjuvant gemcitabine plus S-1 therapy [59]. Additionally, another study reported that the number of Ki-67-positive cells in the tertiary lymphoid organs within the PDAC tumor microenvironment was higher in the neoadjuvant GnP therapy group than in the upfront surgery group [60]. This study also showed significantly higher overall survival rates after resection in the neoadjuvant GnP group compared to the upfront surgery group [60]. In contrast, it was reported that high Ki-67 expression after neoadjuvant therapy has been linked to worse survival in resectable PDAC patients [61]. This apparent contradiction highlights the complex nature of Ki-67 as a prognostic marker in PDAC. It suggests that Ki-67 can be a multifaceted marker, potentially useful for assessing treatment response and predicting outcomes, but requiring further investigation to fully understand its implications. Therefore, a more detailed understanding of the complex cellular responses occurring in residual cells after treatment with the combination of rapamycin and GP is crucial, and further research is needed to elucidate the underlying mechanisms.

In conclusion, despite recent advances, the prognosis of patients with advanced PDAC remains poor, with a 5-year survival rate of 3.1% in the United States [62]. Therefore, there is a pressing need for the development and clinical implementation of new and effective treatment strategies. The combination of rapamycin with GnP therapy holds promise for improving the prognosis of patients with advanced PDAC by leveraging rapamycin’s ability to suppress tumor invasion and GnP’s antiproliferative activity. Additionally, the careful management of side effects may ensure that the quality of life of patients is maintained. We are planning a multicenter, investigator-initiated, phase I/II clinical trial to investigate whether the combination of rapamycin and GnP therapy has a higher response rate than GnP monotherapy in patients diagnosed with metastatic PDAC. If the findings obtained demonstrate that rapamycin enhances the efficacy of GnP therapy, it has potential as a treatment candidate that exhibits superiority over the current standard first-line chemotherapy, GnP therapy, in future phase III trials.

## Data Availability

This clinical trial is currently in the preparation stage and has not yet been conducted. Therefore, no trial results are available for disclosure. The details of the trial plan are confidential and cannot be released.
